# Supplementary material for: Structural manipulations of a shelter resource reveal underlying preference functions in a shell-dwelling cichlid fish
Source: Proc Biol Sci. 2020 May 20;287(1927):20200127. doi: 10.1098/rspb.2020.0127 (PMC7287357; doi:10.1098/rspb.2020.0127)
Supplement: Supplementary Materials [file rspb20200127supp1.doc]

**Supplementary materials for:**

**Structural manipulations of a shelter resource reveal underlying preference functions in a shell-dwelling cichlid fish**

Aneesh P. H. Bose^†^, Johannes Windorfer^†^, Alex Böhm, Fabrizia Ronco, Adrian Indermaur, Walter Salzburger, Alex Jordan (^†^ co-first authors)

**Proceedings of the Royal Society B**

*Fish housing conditions*

Our laboratory trials took place between April 29 – July 13, 2019. *Neolamprologus multifasciatus* individuals were housed in sex-specific aquaria (75 x 90 x 35 cm) when not being used in a choice task. Fish were held at a density of ~ 1 fish per 6 L. The aquaria were lined with ~ 4 cm of aquarium sand. Water was held between 24 – 28°C and pH of 7.6 - 8.6. Fish were not given any *Neothauma tanganyicense* shells or shell replicas while in these holding tanks to avoid giving them recent experience with a particular shell size or type. Instead, they were given open-ended PVC tubes (3 - 6 cm in length) for shelters. Fifty percent water changes were performed once a week. Fish were fed fresh Artemia nauplii and either a frozen mix of Artemia, Mysis and Daphnia or Granules (Naturefood Supreme Artemia Marin - M) once per day. Experimental tanks were held to the same water quality standards, but experimental fish were not fed over the course of their 20 hr trials, though the stimulus groups were fed in between trials. Stimulus groups remained in their acrylic cylinders within the experimental aquaria for the entire duration of the experiment. Doing so allowed the stimulus groups to establish their territories and social relationships without disturbing them with repeated handling between trials. Thus, our design aimed to minimize stress in our study animals and this was evidenced by the stimulus groups displaying their natural repertoire of social behaviours while residing in the cylinders.

*Shell scanning, modelling, and printing*

We scanned an average-sized, fully intact *N. tanganyicense* shell in a Bruker Skyscan 1174v2 CT-Scanner at 50 kV, 800 µA with a 0.5 mm Aluminium filter and 4900ms exposure time and 29.9 µm voxel size. Three to four images were averaged per frame with 0.8° rotation steps in an 180° scan, resulting in 238 projections. Reconstruction was performed using NRecon (v 1.6.10.2), post-processing of scan data was done in Fiji (ImageJ, v 2.0.0) and Meshlab (v 2016-12). The resulting 3D model with 5 million faces was imported into Fusion360 (Autodesk) and compressed to 15000 faces. The model was then transformed, exaggerating or diminishing certain attributes, as needed for the experiment (see Supplementary Table 1). The designed models were sliced (layer thickness 0.08 – 0.12 mm) and printed using Ultimaker Cura with a modified Anet A8 3D-Printer and PETG-Filament.

**Supplementary Materials Figure Captions**

**Supplementary Materials Figure 1**: Example of an open-ended preference function spline, taken from one female *Neolamprologus multifasciatus* in the shell size choice task. The point where the red line meets the x-axis represents peak preference, the slope of the curve as it drops away from the red line represents preference strength, and the width of the blue line represents tolerance.

**Supplementary Materials Figure 2**: Results of shell choice tasks in which (A) overall shell size, (B) shell length, (C) shell aperture width, and (D) shell intactness were manipulated using *Neothauma tanganyicense* shell replicas as described in Methods. Grey density plots illustrate the natural distribution of these attributes as observed in wild-collected shells (N = 113, see Methods). The population density plot for shell size was created by using a principal component analysis to reduce shell length, shell width, and aperture width data from the 113 wild-collected shells into one composite variable, PC1 (accounting for 90.6% of the total variance). There is no comparable population data for shell intactness. The curves are spline fits that represent group-level preference functions for all male (blue) and female (red) *N. multifasciatus* as well as both sexes pooled together (dotted). Splines were generated in the program PFunc [15].

**Supplementary Table 1:** Breakdown of dimensions (in mm) of 3D-printed shell replicas. Shell replicas were printed to represent the full population range for each structural attribute, spanning -3 to +3 SD around the population mean. For the replicas spanning the range of shell intactness, the first two holes closest to the aperture had a diameter of 8 mm, while the other two holes closer to the apex had a diameter of 5 mm. In some replicas, the apex was sometimes cut off at 8 mm to represent shells with broken tips. For the chirality choice trial, mean-sized (0 SD) shell replicas were used, but their chirality was flipped to be ‘left-coiled’.

| *Shell size manipulations* | | | | | | | |
| --- | --- | --- | --- | --- | --- | --- | --- |
| Dimension | -3 SD | -2 SD | -1 SD | 0 SD | +1 SD | +2 SD | +3 SD |
| Shell length | 25.8 | 31.6 | 37.4 | 43.2 | 49.0 | 54.8 | 60.6 |
| Shell width | 20.9 | 24.7 | 28.5 | 32.3 | 36.1 | 39.9 | 43.7 |
| Aperture width | 10.2 | 11.8 | 13.4 | 15.0 | 16.6 | 18.2 | 19.8 |
| 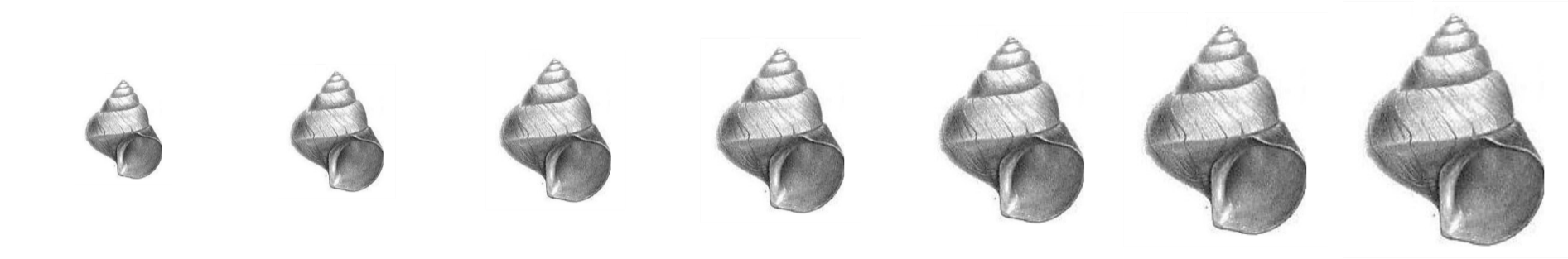 | | | | | | | |
| *Shell length manipulations* | | | | | | | |
| Dimension | -3 SD | -2 SD | -1 SD | 0 SD | +1 SD | +2 SD | +3 SD |
| Shell length | 25.8 | 31.6 | 37.4 | 43.2 | 49.0 | 54.8 | 60.6 |
| Shell width | 32.3 | 32.3 | 32.3 | 32.3 | 32.3 | 32.3 | 32.3 |
| Aperture width | 15.0 | 15.0 | 15.0 | 15.0 | 15.0 | 15.0 | 15.0 |
| 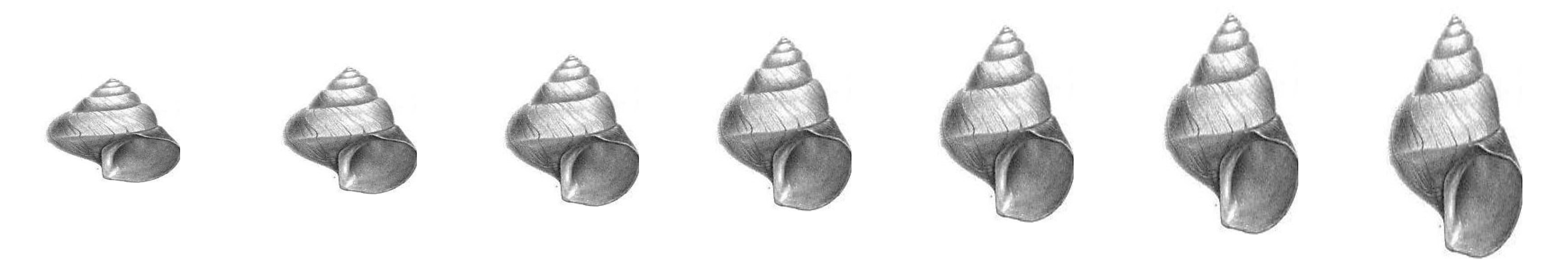 | | | | | | | |

**Supplementary Table 1 continued**

| *Shell aperture manipulations* | | | | | | | |
| --- | --- | --- | --- | --- | --- | --- | --- |
| Dimension | -3 SD | -2 SD | -1 SD | 0 SD | +1 SD | +2 SD | +3 SD |
| Shell length | 43.2 | 43.2 | 43.2 | 43.2 | 43.2 | 43.2 | 43.2 |
| Shell width | 32.3 | 32.3 | 32.3 | 32.3 | 32.3 | 32.3 | 32.3 |
| Aperture width | 10.2 | 11.8 | 13.4 | 15.0 | 16.6 | 18.2 | 19.8 |
| 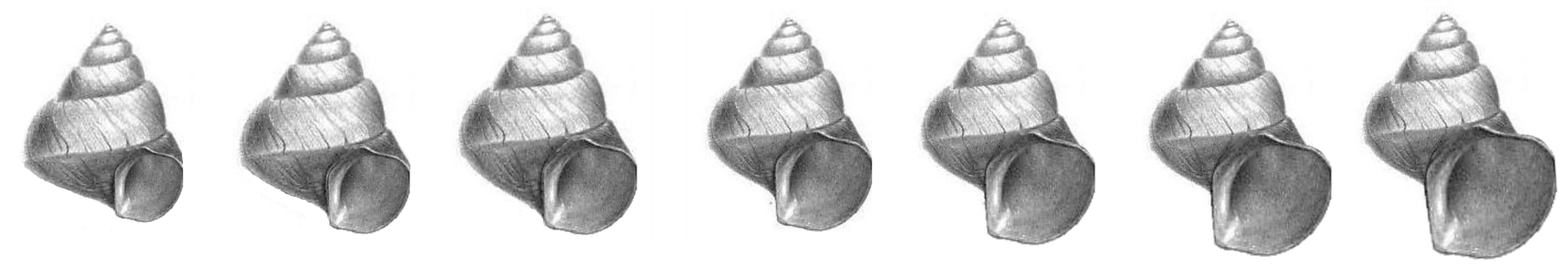 | | | | | | | |
| *Shell intactness manipulations* | | | | | | | |
| Dimension | Fully intact |  |  |  |  |  | Least intact |
| Shell length | 43.2 | 43.2 | 43.2 | 43.2 | 43.2 | 43.2 | 43.2 |
| Shell width | 32.3 | 32.3 | 32.3 | 32.3 | 32.3 | 32.3 | 32.3 |
| Aperture width | 15.0 | 15.0 | 15.0 | 15.0 | 15.0 | 15.0 | 15.0 |
| Number of holes | 0 | 0 | 1 | 2 | 2 | 3 | 4 |
| Tip cut off? | No | Yes | No | No | Yes | No | No |
| 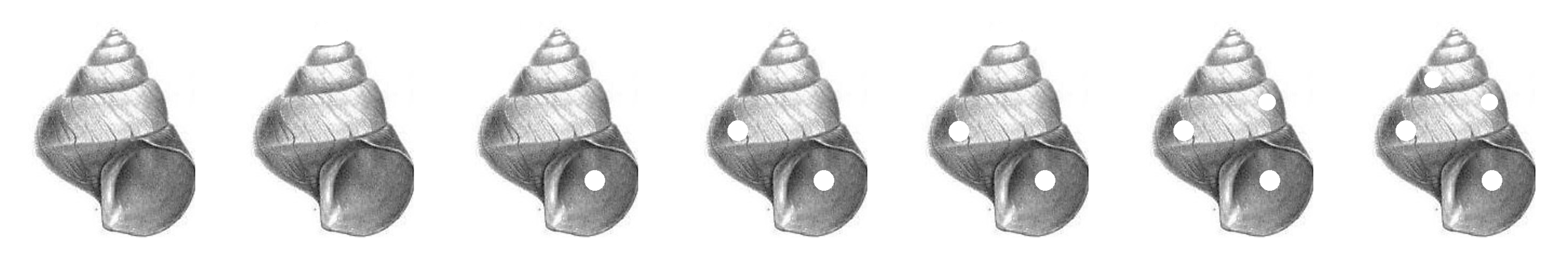 | | | | | | | |
