# Supplementary figures and images for: Structural manipulations of a shelter resource reveal underlying preference functions in a shell-dwelling cichlid fish
Source: Proc Biol Sci. 2020 May 20;287(1927):20200127. doi: 10.1098/rspb.2020.0127 (PMC7287357; doi:10.1098/rspb.2020.0127)

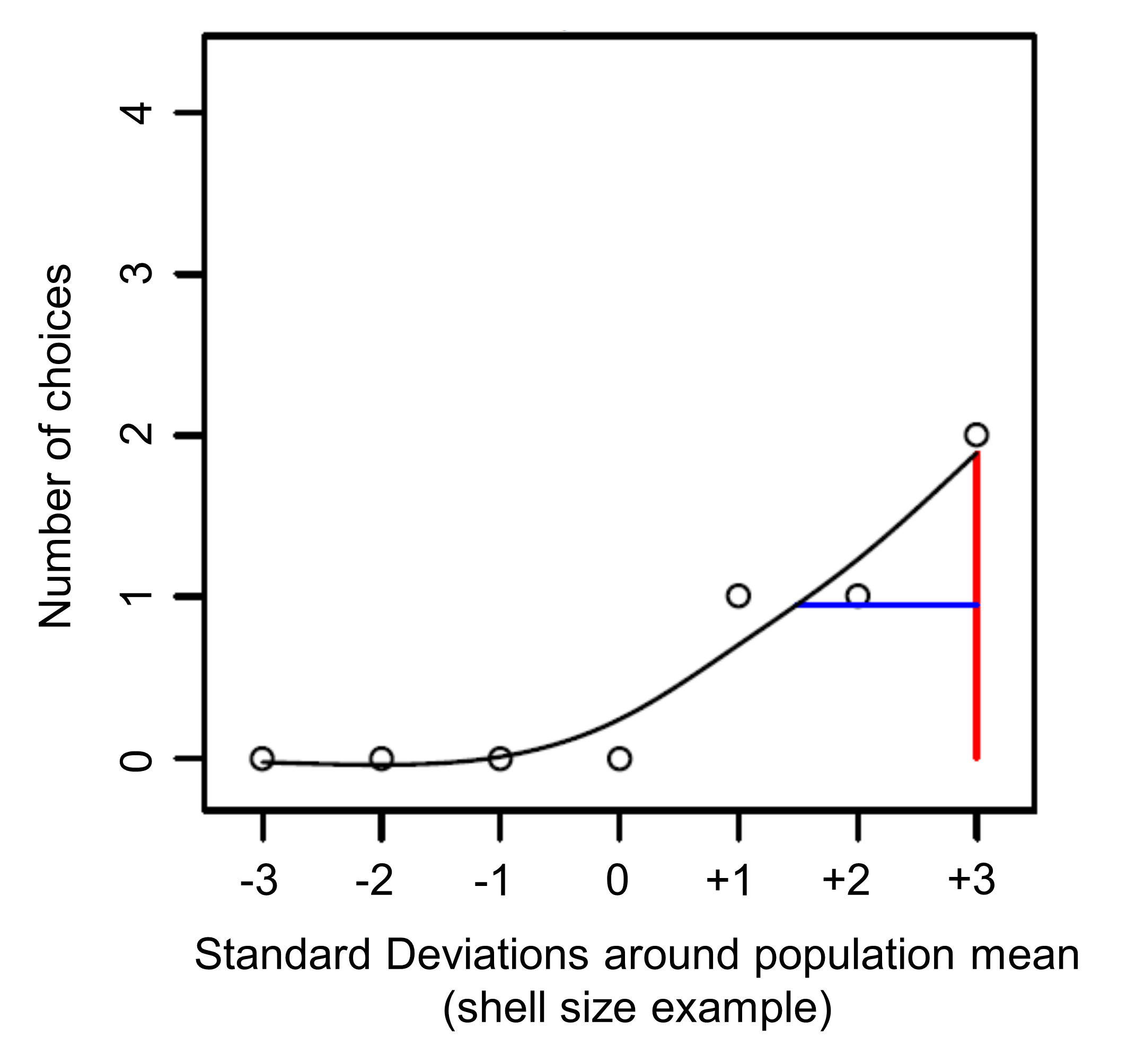

Supplement: Figure S1 [file rspb20200127supp2.tif]

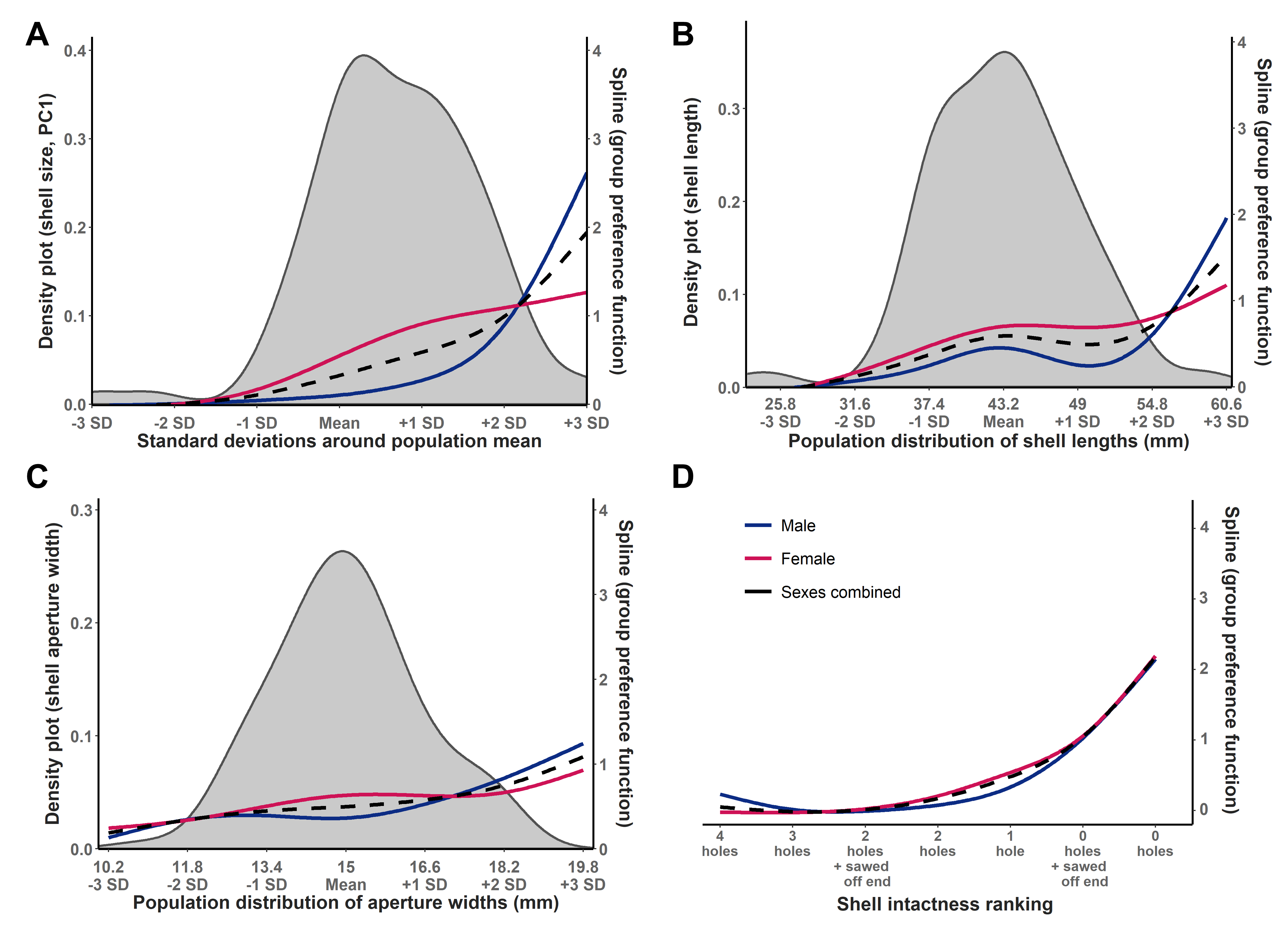

Supplement: Figure S2 [file rspb20200127supp3.tif]
